# Reducing private health expenditure in Greece: the case for regulated complementary health insurance

**DOI:** 10.3389/fpubh.2026.1810695

**Published:** 2026-04-10

**Authors:** Stefanos Karakolias, Miltiadis Nektarios, Panos Xenos, Nikolaos Polyzos

**Affiliations:** 1 Department of Nursing, Democritus University of Thrace, Alexandroupolis, Greece; 2 Department of Statistics and Insurance Science, University of Piraeus, Piraeus, Greece; 3 Department of Medicine, Democritus University of Thrace, Alexandroupolis, Greece

**Keywords:** complementary health insurance, financial protection, Greece, health financing, health system regulation, out-of-pocket payments, private health expenditure

## Abstract

Greece records the highest level of private health expenditure in the European Union, driven largely by out-of-pocket payments that undermine financial protection and exacerbate inequities. Despite universal coverage, benefit gaps, cost-sharing arrangements, and limits in publicly financed service availability shift a substantial financial burden onto households. Within a dual insurance system, voluntary private health insurance frequently overlaps with public benefits and does little to reduce excessive out-of-pocket spending. A regulated complementary health insurance framework, embedded within a three-tier financing structure, could convert part of private spending into pooled and prepaid contributions while preserving the primacy of public coverage. Targeted regulatory, institutional, and design reforms are therefore proposed to ensure that complementary insurance supports equity-oriented health financing rather than reinforcing disparities.

## 1 Introduction

Private health expenditure, particularly out-of-pocket (OOP) payments, remains a central challenge for health system performance and financial protection across Europe (1, 2). High reliance on direct household payments exposes individuals to unpredictable costs and may limit access to care (3). In systems with near-universal public coverage, persistent private spending signals structural gaps rather than lack of entitlement (4). Although voluntary private insurance has expanded as a supplementary mechanism, its impact on reducing OOP exposure has been modest and unevenly distributed (5, 6). This raises a central policy question: can private insurance be reshaped to support, rather than undermine, equity-oriented health financing?

Greece represents a particularly instructive case. Despite universal public health insurance coverage, households continue to finance a substantial share of health care costs directly. This pattern has important equity implications, as the burden falls unevenly across income groups and is associated with catastrophic health spending and unmet health care needs (7–9). These outcomes reflect longstanding structural weaknesses, compounded by fragmented policy responses and limited monitoring capacity (10), alongside more recent pressures such as fiscal constraints, cost-sharing arrangements, and limits in publicly financed service availability (7, 11). Concurrently, population aging and the growing prevalence of chronic conditions increase demand for services that often entail repeated and cumulative OOP costs (12). Together, these trends raise questions about the system’s ability to deliver universal health coverage with effective financial protection and equitable resource allocation (13).

This policy brief evaluates the case for introducing regulated complementary health insurance as a structural reform to reduce private health expenditure in Greece. Rather than treating private financing as a residual or unavoidable outcome, the paper focuses on how it is structured, governed, and integrated with public coverage. While Greece is used as a primary case due to the magnitude of its private expenditure, the policy considerations discussed are relevant to other health systems confronting persistent gaps between statutory coverage and effective financial protection.

## 2 Problem statement and context

**Figure 1:**
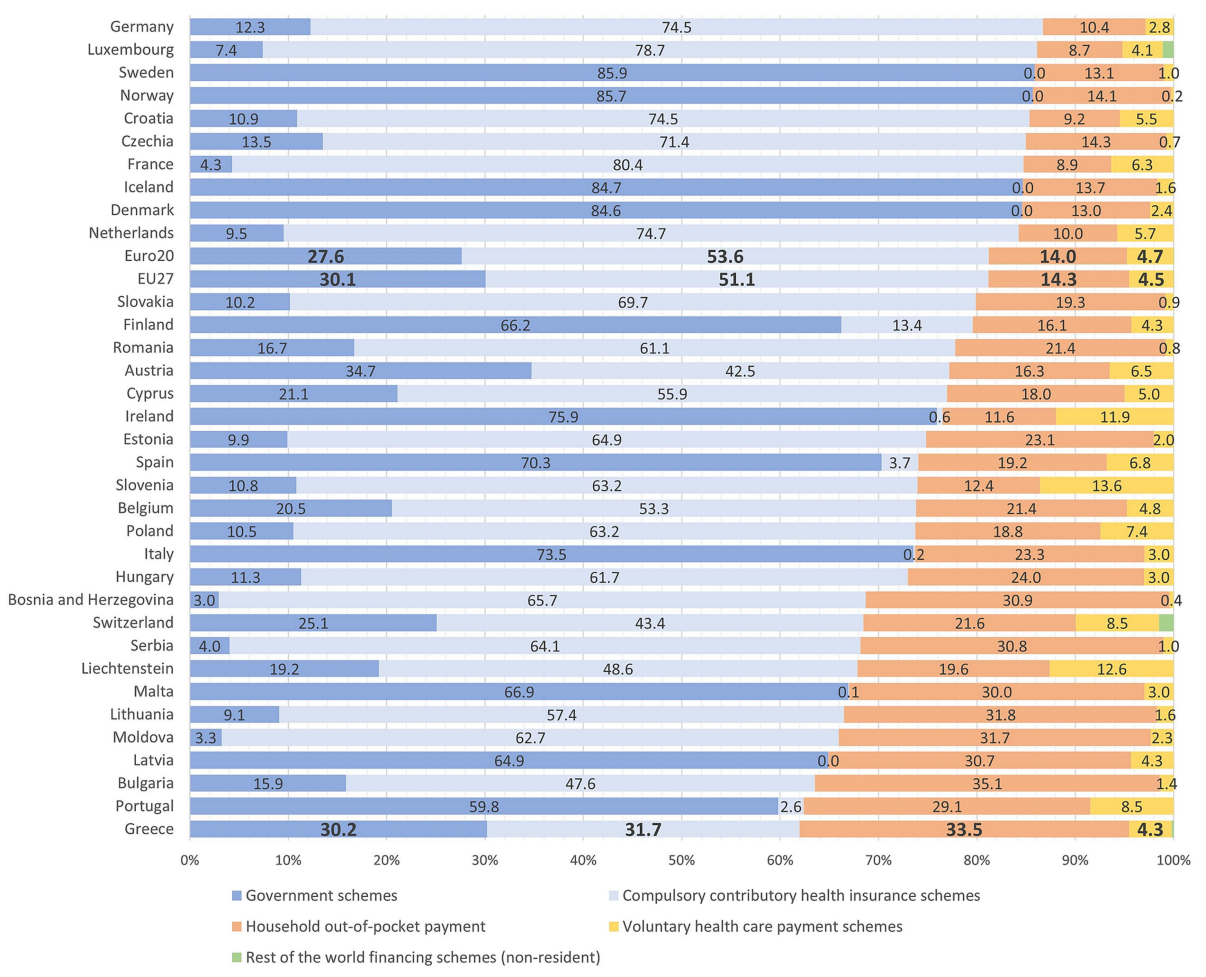
Sources of health expenditure financing in Europe, 2022. Source: Authors’ calculations based on Eurostat data (34).

Greece stands out among European health systems for the exceptionally large role that households play in financing health care, placing it outside the dominant Beveridge- and Bismarck-type models observed across Europe. As shown in Figure 1, OOP payments account for approximately 33.5% of total health expenditure in Greece—more than double the EU27 and Euro20 averages of around 14%. This divergence reflects the comparatively weak contribution of social health insurance, which finances a markedly smaller share of total expenditure than in most European systems, by roughly 20 percentage points. Instead of being absorbed through stronger public financing or expanded risk pooling, the approximately 20-percentage-point financing gap is transferred to households, with OOP payments functioning as the de facto mechanism through which coverage gaps are financed at the individual level. When household-financed private insurance premiums—amounting to a further 4.3% of total expenditure—are also considered, Greece records the highest overall private share of health spending among EU countries.

Private health insurance in Greece operates alongside mandatory public coverage within a dual insurance arrangement. While all residents are compulsorily insured through the unified public purchaser (EOPYY), private insurance is purchased voluntarily and frequently overlaps with services formally included in the public benefit package. In practice, however, this overlap does not translate into systematic reduction of OOP spending. Instead, private coverage primarily enhances access—by facilitating shorter waiting times, greater provider choice, or improved service conditions—rather than functioning as a complementary risk-pooling mechanism targeting statutory cost-sharing or defined coverage gaps. As a result, households may finance similar entitlements twice, yet the principal drivers of private expenditure—particularly OOP payments for pharmaceuticals, hospital care, and ambulatory services—remain largely unaffected (Supplementary Table 1).

**Figure 2:**
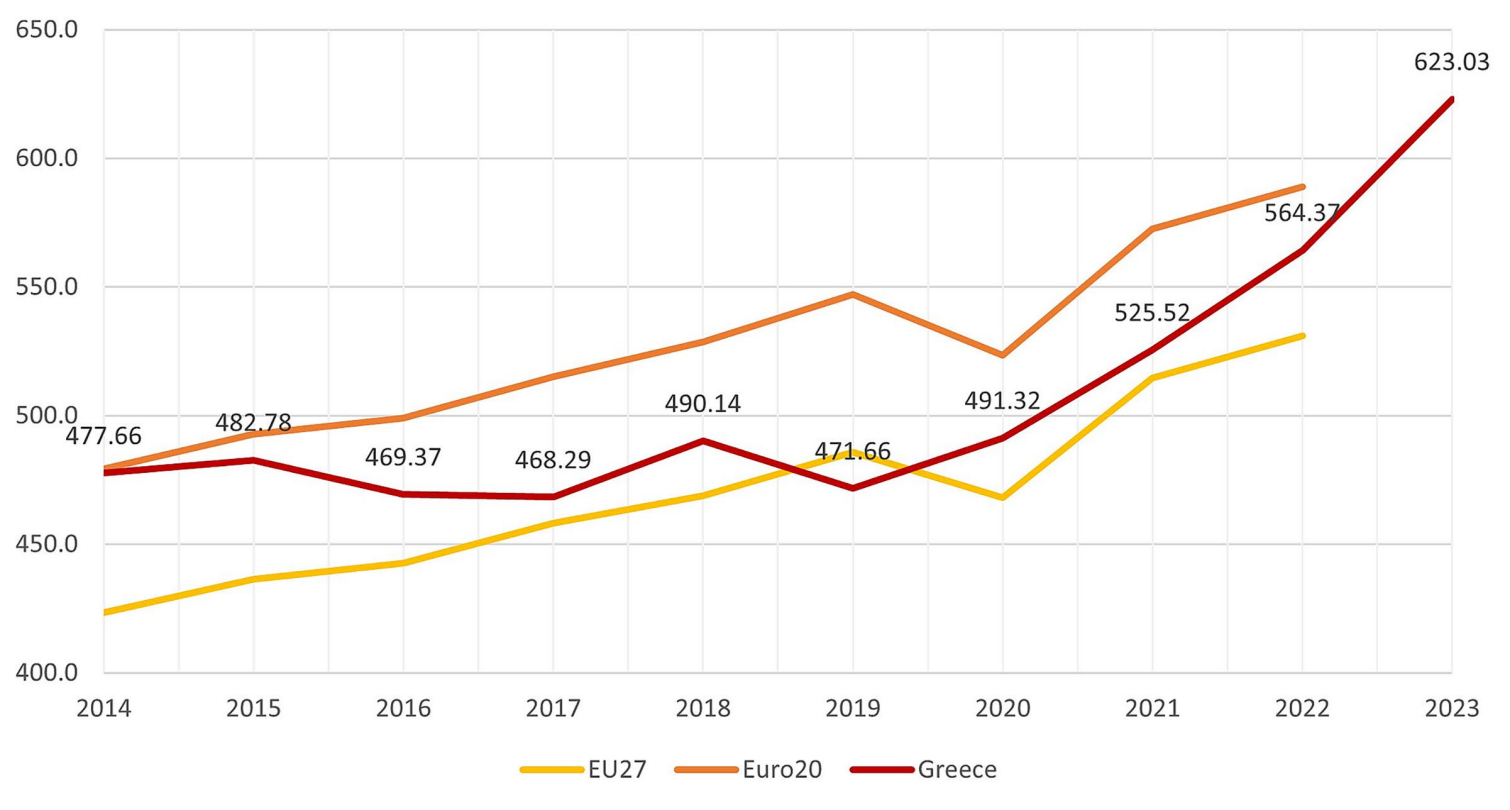
Out-of-pocket health payments per capita in Greece and Europe, 2014–2023 (EUR). Source: Authors’ calculations based on Eurostat data (34).

The persistence of this pattern is illustrated in Figure 2, which shows that per capita OOP spending in Greece has consistently exceeded EU27 and Euro20 averages throughout 2014–2023, surpassing €600 per capita in recent years. This sustained divergence indicates that reliance on household financing is structural rather than a temporary response to fiscal shocks or short-term policy choices.

Incremental adjustments within the existing framework are unlikely to substantially reduce private spending. Population aging in Greece is accelerating, and health expenditure is increasingly concentrated among individuals aged 65 and over, whose utilization of hospital and pharmaceutical services is predictably higher. Without structural reform, rising demand is likely to translate into continued reliance on direct household payments. At the same time, persistent fiscal constraints limit the short- to medium-term scope for expanding publicly financed benefits. Under these conditions, the persistent use of OOP payments to offset financing gaps becomes increasingly unsustainable, with adverse consequences for equity and financial protection. The present moment therefore represents a critical window to reorganize private health financing in a way that strengthens risk pooling rather than reinforces segmentation. These considerations underscore the need for a more deliberate policy approach, examined in the following section.

## 3 Policy options analysis

Before assessing the three options, it is important to acknowledge that expanding publicly financed coverage represents, in principle, the most direct mechanism for strengthening financial protection. Increasing statutory benefits, reducing cost-sharing, or raising public health expenditure would directly lower reliance on OOP payments and enhance equity. However, in the Greek context, persistent fiscal constraints, high public debt levels, and competing budgetary pressures limit the short- to medium-term feasibility of substantial expansion in public health financing. Moreover, demographic aging is projected to increase baseline expenditure needs, making large-scale benefit expansion fiscally demanding. Under these conditions, the policy question is not whether stronger public financing is desirable, but how to reorganize existing private expenditure in a manner consistent with solidarity principles while preserving the primacy of statutory coverage.

Addressing the high level of private health expenditure in Greece requires a deliberate policy choice regarding the role of private financing within the broader health system. In practice, three policy options can be identified: continuation of the current financing structure, expansion of private health insurance through market forces, and the introduction of a regulated complementary insurance framework integrated with public coverage. Each option carries distinct implications for financial protection, equity, and system sustainability.

The three policy options are assessed using a set of explicit evaluation criteria derived from health financing principles. These include: (i) impact on financial protection, particularly the reduction of OOP payments; (ii) distributional consequences and implications for equity; (iii) the extent to which each option strengthens or weakens risk pooling; (iv) fiscal sustainability within prevailing budgetary constraints; and (v) political and institutional feasibility. Applying these criteria allows for a structured comparison rather than a purely descriptive presentation of alternatives.

### 3.1 Option 1: continuation of the status quo

Under the status quo, the existing financing structure remains largely unchanged. Private health expenditure—primarily OOP payments—continues to compensate for coverage gaps and service limitations in the public system, while voluntary private insurance develops organically without strategic coordination with statutory coverage. In this scenario, the state neither actively restructures private financing nor deliberately promotes its expansion; instead, fragmentation persists as an equilibrium outcome of institutional inertia.

Evidence from Greece indicates that this arrangement sustains a high and uneven financial burden on households (14). With OOP already accounting for an exceptionally large share of total health expenditure, risks of financial hardship and unmet need remain substantial. While private insurance may continue to grow incrementally in response to access constraints, its expansion under this passive model does not systematically target cost-sharing obligations or defined coverage gaps. Maintaining the current structure therefore risks entrenching household risk exposure and deepening inequities.

### 3.2 Option 2: market-driven expansion of private health insurance

A second option entails a deliberate policy stance favoring the expansion of private health insurance through market mechanisms. Under this approach, the state refrains from imposing strong regulatory coordination with statutory coverage and instead allows private insurers to expand product offerings in response to demand, with premiums reflecting individual risk profiles and benefit packages shaped primarily by market competition.

While private insurance in Greece is already growing without explicit public-sector endorsement, this option differs from the status quo in that it implicitly relies on market expansion as the principal mechanism for addressing public system gaps. In practice, such growth is likely to concentrate among higher-income groups, as voluntary uptake remains linked to ability to pay. International evidence suggests that in the absence of structured pooling requirements and benefit standardization, market-driven expansion has limited impact on aggregate OOP spending and may reinforce socioeconomic gradients in access (15, 16). Although this approach may ease fiscal pressure on the public sector in the short term, it does so by increasing system segmentation rather than strengthening financial protection at the population level.

### 3.3 Option 3: regulated complementary health insurance

The third option entails the introduction of a regulated complementary health insurance framework explicitly designed to complement public coverage. Under this model, complementary insurance targets specific components of private expenditure—such as copayments, deductibles, and services not fully covered by the public system—within a clearly defined regulatory and institutional framework. Core design features include risk pooling, benefit standardization, and coordination with the public purchaser, ensuring that complementary coverage fills predefined gaps rather than substituting public entitlements (17, 18).

Evidence from international experience suggests that regulated complementary insurance can reduce OOP payments by converting unpredictable private spending into pre-paid and pooled contributions, thereby lowering exposure to catastrophic health expenditure (16). European experience illustrates this potential. In France, complementary insurance covering statutory cost-sharing is associated with an OOP share of approximately 9% of total health expenditure, among the lowest in the EU, alongside comparatively low levels of catastrophic and impoverishing spending (19). In Slovenia, where complementary coverage for copayments is widespread, OOP accounts for roughly 12–13% of total expenditure, below the EU average, with very low reported rates of catastrophic spending among lower-income households (20). Croatia similarly records OOP levels of approximately 14–15% of total expenditure, with complementary insurance playing a role in limiting financial hardship, although voluntary uptake without strong redistributive safeguards may reproduce socioeconomic gradients (21).

Taken together, these cases indicate that complementary insurance contributes positively to financial protection only when strongly regulated. International evidence indicates that complementary insurance improves financial protection only under structured regulatory conditions (18, 22). When appropriately designed, regulated complementary insurance can enhance financial protection—particularly for older individuals and households with chronic health needs—while safeguarding the central role of statutory public insurance (17).

### 3.4 Policy feasibility and comparative assessment

Applying the stated evaluation criteria—financial protection, equity, risk pooling, fiscal sustainability, and institutional feasibility—the three options produce clearly differentiated outcomes.

Under the status quo, high OOP spending remains structurally embedded, sustaining household financial risk and uneven access. Market-driven expansion of private insurance may reduce financial exposure for those able to purchase coverage, but its voluntary and risk-rated nature limits population-level reductions in OOP and tends to reinforce socioeconomic gradients (23).

By contrast, regulated complementary insurance directly targets predefined cost-sharing components and coverage gaps, converting part of private expenditure into pooled and prepaid contributions. If combined with pooling requirements and affordability safeguards, this approach is more consistent with equity principles in mixed financing systems (24, 25), while preserving the primacy of statutory coverage. To mitigate potential crowd-out of statutory coverage, rollout should be accompanied by explicit commitments to maintain or strengthen Tier I benefits and by periodic review of cost-sharing levels within the public package.

Implementation nevertheless requires careful design. In Greece, private insurance has evolved largely without coordination with statutory coverage, and public trust in private insurers remains limited. Gradual implementation, strong regulatory oversight, and clear delineation between public and complementary benefits are therefore essential.

Although Greece is an outlier in terms of OOP magnitude, the underlying drivers—cost-sharing arrangements, partial coverage, fiscal constraints, and demographic pressures—are not unique. The framework’s transferability depends less on the absolute level of OOP and more on whether private spending is concentrated in predictable, policy-defined components that can be pooled. Greece thus functions as a stress-test case: if high and fragmented household payments can be converted into regulated pooling under these conditions, similar design principles may apply to systems facing persistent financial protection gaps.

## 4 Actionable recommendations

Based on the comparative assessment and available evidence, the following recommendations are proposed. In the short term, priority should be given to regulatory clarification and benefit definition, while more complex elements—such as risk adjustment, affordability safeguards, and coordination mechanisms—can be phased in as institutional capacity and regulatory experience strengthen.

### 4.1 Establish a national regulatory framework for complementary health insurance

A national regulatory framework should clearly define complementary health insurance as a complement to public coverage rather than a substitute for it (15). While the Greek regulatory environment already provides a basic foundation for insurance supervision, it has not been systematically used to align complementary insurance with public financing objectives. Institutionally, the complementary component could be administered either by the public purchaser (EOPYY) or by licensed private insurance companies, operating under a framework of regulated competition and actuarial oversight. Under such an arrangement, complementary coverage would remain voluntary but subject to standardized benefit definitions, pooling requirements, and supervisory oversight. Irrespective of whether the administrator is public or private, regulation should prohibit risk-based exclusions, ensure transparent premium-setting, and require alignment with the statutory benefit package.

Beyond formal supervision, effective implementation requires enforceable regulatory instruments. These may include mandatory product registration and approval procedures, standardized reporting of claims and premium data, and legally binding coordination agreements between insurers and the public purchaser. Insurers offering complementary coverage should be required to submit periodic administrative datasets to a designated supervisory authority, enabling oversight of pricing practices, risk selection patterns, and coverage boundaries. Sanctions for non-compliance—such as administrative fines proportionate to premium volume, suspension or withdrawal of product authorization, exclusion from contracting with publicly funded providers, or mandatory restitution to insured individuals—should be explicitly codified within the supervisory framework. Given the relatively low level of institutional trust in Greece, regulatory credibility depends not only on rule design but also on visible enforcement capacity, potentially through an independent supervisory body or strengthened actuarial oversight within the existing insurance regulatory authority. In the Greek context, supervisory responsibility could be assigned to the existing insurance supervisory authority with dedicated health-insurance capacity, or to a formally designated independent unit mandated to oversee complementary products in coordination with the Ministry of Health (MoH) and EOPYY.

Evidence suggests that regulation should explicitly delineate the boundaries between public and complementary coverage by specifying eligible benefits, such as copayments, deductibles, and services not fully reimbursed by the public system (26). Standardized benefit packages would improve transparency, limit unnecessary duplication, and facilitate coordination with the public purchaser. In parallel, regulatory provisions should require risk pooling mechanisms and prohibit practices that undermine solidarity, such as risk-based exclusions or selective underwriting.

Effective regulatory implementation must also include market-conduct safeguards, such as monitoring premium levels, enforcing clear disclosure requirements, and establishing mechanisms for periodic review of complementary products in relation to statutory benefit design (27). Strengthening regulation does not primarily require new legislation, but rather a reorientation of existing supervisory capacity toward financial protection and equity objectives.

### 4.2 Target high OOP components through benefit design

OOP payments in Greece are concentrated in a limited set of cost components, notably copayments and deductibles for covered services, pharmaceutical costs for uncovered medications, and services with partial or no public reimbursement. A core design principle of complementary insurance is therefore to prioritize coverage of these high-burden components (28). By targeting the main drivers of OOP spending, complementary insurance can strengthen financial protection and reduce exposure to unpredictable health care costs. Benefit packages should be standardized to a core minimum in order to enhance transparency, prevent unnecessary duplication, and facilitate coordination with statutory coverage, while allowing limited flexibility for optional extensions.

### 4.3 Introduce risk-pooling and anti-selection mechanisms

To prevent adverse selection and exclusion of high-risk individuals, complementary insurance must incorporate risk-pooling mechanisms (29). These may include age-adjusted contributions, cross-subsidization rules, or collective enrollment arrangements linked to employment or social insurance status. The fundamental principle must be that insurance operates as a risk-pooling mechanism in which the insurer commits to provide continuous coverage and compensation throughout the period of insurance validity. Without such mechanisms, complementary insurance risks becoming inaccessible to older individuals and those with chronic conditions, thereby undermining its financial protection role. Where voluntary uptake proves insufficient to sustain broad pooling, implementation could consider default enrollment with opt-out provisions or group-based enrollment channels (e.g., through pension funds or occupational schemes), combined with targeted subsidies for low-income groups to preserve equity.

### 4.4 Ensure affordability safeguards for vulnerable populations

Affordability measures should be embedded in the design of complementary insurance to protect low-income households and older adults (30). Policy tools may include income-related subsidies, partial public co-financing, premium caps for defined population groups, or the introduction of income-based cost-sharing arrangements in place of flat-rate structures (31). Such measures are critical to prevent complementary insurance from functioning as a regressive financing mechanism and to align it with equity objectives. The regulatory framework must establish that no exclusions or waiting periods can be applied based on pre-existing conditions.

### 4.5 Strengthen coordination between public and private insurers

Effective implementation requires structured coordination between the public purchaser and private insurers. In several European health systems, this is achieved through data-sharing protocols, aligned reimbursement rules, and clearly defined interfaces between public benefits and complementary coverage, which help prevent fragmentation and inefficiencies (32). Operationally, this coordination may include standardized exchange of anonymized claims-level data (benefit category, provider type, cost-sharing component, paid amounts), harmonized coding of reimbursable services, and predefined rules governing the sequencing of statutory and complementary reimbursement. Such coordination enhances system transparency, strengthens monitoring of private expenditure flows, and supports more efficient purchasing of health services. Experience in Greece also shows that formal collaboration frameworks can be established through explicit cooperation agreements between public health system entities and private insurers, supported by clear pricing rules and coordinated benefit design.

### 4.6 Implement monitoring and evaluation mechanisms focused on financial protection

The impact of complementary insurance should be evaluated through systematic monitoring of financial protection indicators, including trends in OOP expenditure, catastrophic spending incidence, and unmet need across income and age groups (33). Monitoring should be institutionally separated from regulatory enforcement and embedded within the MoH or an independent evaluation body. Periodic public reporting would enhance transparency and allow policy recalibration where unintended segmentation or inequitable access patterns emerge. Evidence-based evaluation strengthens accountability and supports adaptive reform over time.

## 5 Implementation framework: a three-tier financing structure

**Figure 3:**
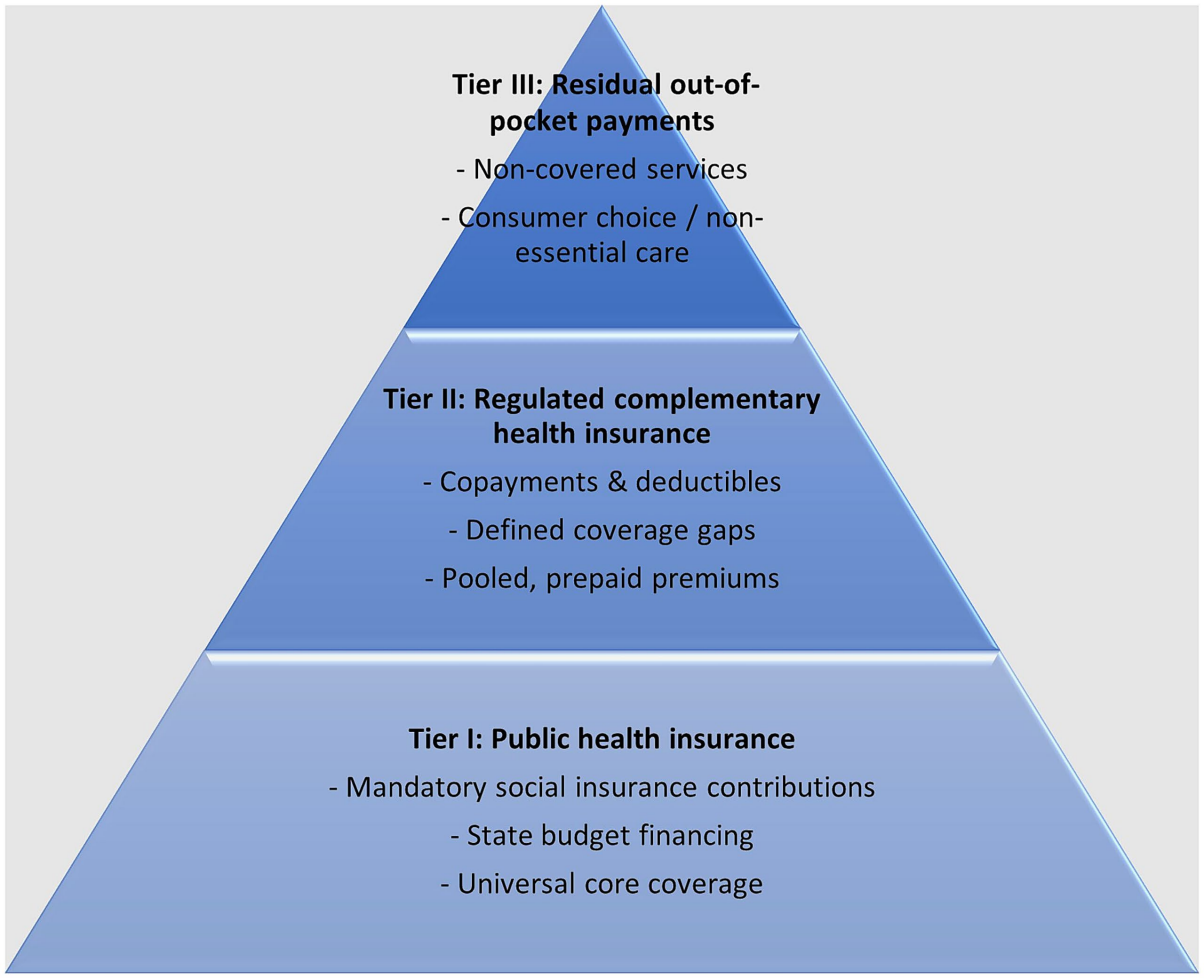
Three-tier health financing structure integrating regulated complementary insurance. Source: Authors’ own work.

Operationally, the reform can be implemented through a three-tier health financing structure that clearly delineates the roles of public coverage, regulated complementary insurance, and residual OOP payments (Figure 3). Tier I consists of publicly financed statutory insurance providing universal access to a defined core package of services. Tier II introduces regulated complementary insurance designed to cover predefined coverage gaps, including copayments, deductibles, and services not fully reimbursed under statutory benefits. Tier III comprises residual OOP payments for services falling outside both statutory and complementary coverage. This architecture reorganizes existing private expenditure into pooled contributions within a statutory framework. Detailed characteristics of each tier are presented in Supplementary Table 2. Implementation should proceed in phased stages, beginning with regulatory clarification and benefit standardization, followed by progressive integration of pooling and affordability mechanisms. The framework is intended to reallocate a defined portion of recurrent OOP components into pooled contributions; the magnitude of any reduction in aggregate OOP will depend on the specific services included in the standardized complementary package and on uptake rates. These parameters should be refined through pilot implementation and claims-based modeling prior to national rollout.

A central design issue within Tier II concerns the pooling of predictable age-related expenditure. Individuals aged 65 and over account for a disproportionately large share of total health spending relative to their population size, reflecting systematically higher per capita utilization of hospital and pharmaceutical services (Table 1). To illustrate how such expenditure patterns could be pooled within a regulated complementary framework—rather than to propose actuarially calibrated premiums—Table 1 presents indicative contribution ranges derived proportionally from aggregate age-related expenditure shares. The euro ranges are scaled to approximate current per-capita private spending levels and are used solely to illustrate relative contribution gradients across age groups; they do not constitute actuarial premium estimates.

**Table 1:** Illustrative distributional scenario for age-adjusted complementary contributions.

Age group	Approximate share of total expenditure	Indicative complementary contribution range
<65	~60%	~600 €
65–79	~30%	1.000–1.100 €
80+	~10%	>1.200 €

Contribution ranges are illustrative and based on proportional allocation of aggregate age-related expenditure shares. They are not actuarially determined premium estimates.

For illustrative purposes, a baseline contribution could apply to individuals under 65, with progressively higher contribution ranges for older age groups reflecting predictable differences in utilization. The objective of this graduated structure is not to replicate individual risk-rating, but to pool age-related expenditure transparently within a solidarity-based framework. Without structured pooling, older individuals are more likely to face either unaffordable voluntary premiums or continued exposure to direct OOP payments.

To prevent regressive effects, age-related differentiation must be combined with affordability safeguards, including income-related subsidies, public co-financing of premiums for low-income pensioners, or income-linked contribution caps. These mechanisms aim to ensure broad participation and prevent complementary insurance from reinforcing socioeconomic segmentation. The framework therefore seeks to integrate existing private demand into a regulated structure aligned with statutory coverage, rather than fragment it further.

In summary, the three-tier model reorganizes existing private expenditure into more predictable and pooled financing streams while preserving the central role of public insurance. It does not substitute for strengthening statutory coverage; indeed, expansion of publicly financed benefits remains the most direct route to improving financial protection. However, given persistent fiscal constraints and demographic pressures, complementary insurance may represent a pragmatic mechanism for enhancing financial protection in the short to medium term. Its effectiveness depends critically on strong regulation, enforceable oversight, affordability safeguards, and continuous monitoring of distributional outcomes.

## 6 Conclusion

This policy brief argues that Greece’s exceptionally high reliance on OOP payments is not an unavoidable consequence of fiscal constraint, but the result of how private financing is structured and governed. Maintaining the current model will continue to shift financial risk onto households, disproportionately affecting older individuals and those with chronic conditions. Market-driven expansion of voluntary private insurance, in the absence of strong regulation, is unlikely to reduce population-level OOP spending and risks deepening socioeconomic segmentation.

In the current fiscal environment, rapid expansion of publicly financed benefits may be constrained. Under these conditions, regulated complementary health insurance represents the most feasible structural intervention for reorganizing private health expenditure. By converting predictable cost-sharing and uncovered service payments into pooled and prepaid contributions, a regulated complementary framework can strengthen financial protection while maintaining the foundational role of universal statutory insurance. However, its effectiveness depends critically on enforceable regulation, explicit coordination with the public purchaser, affordability safeguards for vulnerable populations, and continuous monitoring of equity outcomes. Complementary insurance must operate within solidarity-based pooling arrangements rather than substitute for public financing. If appropriately designed and supervised, the proposed three-tier structure offers a pragmatic pathway to reduce household financial exposure in Greece and other health systems facing similar demographic and fiscal pressures.
