# Supplementary material for: Reducing private health expenditure in Greece: the case for regulated complementary health insurance
Source: Front Public Health. 2026 Apr 10;14:1810695. doi: 10.3389/fpubh.2026.1810695 (PMC13106488; doi:10.3389/fpubh.2026.1810695)
Supplement: Supplementary file 1 [file Table_1.DOCX]

Supplementary Material

**Supplementary Table 1.** Distribution of private health expenditure in Greece by provider and financing source, 2023 (€ million).

| **Providers** | | **Voluntary Health Insurance** | **Out-of-pocket payments** | **Total private health expenditure** |
| --- | --- | --- | --- | --- |
| Hospitals | | 628.50 | 2,198.02 | 2,826.52 |
|  | General hospitals | 628.50 | 1,725.67 | 2,354.17 |
|  | Mental health hospitals | 0.00 | 129.08 | 129.08 |
|  | Specialised hospitals (other than mental health hospitals) | 0.00 | 343.27 | 343.27 |
| Residential long-term care facilities | | 0.00 | 0.00 | 0.00 |
|  | Long-term nursing care facilities | 0.00 | 0.00 | 0.00 |
|  | Mental health and substance abuse facilities | 0.00 | 0.00 | 0.00 |
|  | Other residential long-term care facilities | 0.00 | 0.00 | 0.00 |
| Providers of ambulatory health care | | 68.88 | 1,614.25 | 1,683.13 |
|  | Medical practices | 68.88 | 772.40 | 841.27 |
|  | Dental practice | 0.00 | 735.10 | 735.10 |
|  | Other health care practitioners | 0.00 | 106.75 | 106.75 |
|  | Ambulatory health care centres | 0.00 | 0.00 | 0.00 |
|  | Providers of home health care services | 0.00 | 0.00 | 0.00 |
| Providers of ancillary services | | 0.00 | 205.87 | 205.87 |
|  | Providers of patient transportation and emergency rescue | 0.00 | 0.00 | 0.00 |
|  | Medical and diagnostic laboratories | 0.00 | 205.87 | 205.87 |
|  | Other providers of ancillary services | 0.00 | 0.00 | 0.00 |
| Retailers and other providers of medical goods | | 0.00 | 2,465.93 | 2,465.93 |
|  | Pharmacies | 0.00 | 2,414.27 | 2,414.27 |
|  | Retail sellers and other suppliers of durable medical goods and medical appliances | 0.00 | 51.65 | 51.65 |
|  | All other miscellaneous sellers and other suppliers of pharmaceuticals and medical goods | 0.00 | 0.00 | 0.00 |
| Providers of preventive care | | 0.00 | 0.00 | 0.00 |
| Providers of health care system administration and financing | | 108.26 | 0.00 | 108.26 |
|  | Government health administration agencies | 0.00 | 0.00 | 0.00 |
|  | Social health insurance agencies | 0.00 | 0.00 | 0.00 |
|  | Private health insurance administration agencies | 108.26 | 0.00 | 108.26 |
|  | Other administration agencies | 0.00 | 0.00 | 0.00 |
| Rest of economy | | 0.00 | 0.00 | 0.00 |
|  | Households as providers of home health care | 0.00 | 0.00 | 0.00 |
|  | All other industries as secondary providers of health care | 0.00 | 0.00 | 0.00 |
| Rest of the world | | 12.83 | 0.00 | 12.83 |
| Providers unknown | | 0.00 | 0.00 | 0.00 |
|  | **Total** | **818.47** | **6,484.07** | **7,302.54** |

Source: Authors’ calculations based on Eurostat data (14).

**Supplementary Table 2.** Detailed description of the proposed three-tier health financing structure.

| **Tier** | **Tier I** | **Tier II** | **Tier III** |
| --- | --- | --- | --- |
| **Financing source** | Mandatory social insurance contributions | State budget grant to public insurance | Complementary health insurance (public or private) |
| **Population covered** | Employed individuals (~4 million) and pensioners (~2 million) | Entire population | Voluntary uptake |
| **Contribution / premium logic** | Contributions based on wages and pensions, equivalent to a social insurance premium | State transfers treated as an implicit per capita “premium” paid to the public insurance body | Residual premium covering insurable costs beyond Tiers I and II, net of copayments |
| **Approximate per capita amount** | ~€800 per capita annually (≈6% of average gross wage of ~€1,300) | ~€800 per capita annually (comparable to contribution revenues) | ~€600 per capita annually (with copayments of ~€300, corresponding to 20–33% of residual costs) |
| **Role in system financing** | Primary insurance component and main source of financing for health services | Complements Tier I to ensure stable and adequate public financing and avoid premium duplication | Covers predefined gaps, reduces out-of-pocket exposure, and allows additional choice |
| **Key governance features** | Mandatory participation; solidarity-based risk pooling; income-related contributions | Budgetary allocation; transparency of financial flows; reinforcement of universal coverage | Actuarial oversight; regulated benefit design; age-based differentiation; coordination with public coverage |
